# Associations of Maternal Vitamin D Deficiency with Pregnancy and Neonatal Complications in Developing Countries: A Systematic Review

**DOI:** 10.3390/nu10050640

**Published:** 2018-05-18

**Authors:** Paige van der Pligt, Jane Willcox, Ewa A. Szymlek-Gay, Emily Murray, Anthony Worsley, Robin M. Daly

**Affiliations:** 1 Institute for Physical Activity and Nutrition (IPAN), School of Exercise and Nutrition Sciences, Deakin University, Geelong 3220, Australia; ewa.szymlekgay@deakin.edu.au (E.A.S.-G.); anthony.worsley@deakin.edu.au (A.W.); robin.daly@deakin.edu.au (R.M.D.); 2 School of Exercise and Nutrition Sciences, Deakin University, Burwood 3125, Australia; jwillcoxresearch@gmail.com (J.W.); e.murray@deakin.edu.au (E.M.); 3 School of Allied Health, College of Science, Health and Engineering, La Trobe University, Bundoora 3083, Australia

**Keywords:** vitamin D, pregnancy, obstetric, maternal, neonatal, developing countries, pre-eclampsia, gestational diabetes mellitus, low birth weight, small for gestational age

## Abstract

Pregnant women in Asia, the Middle East, Africa and Latin America are at risk of vitamin D deficiency (VDD) and prevalence throughout these regions are among the highest, globally. Maternal VDD has been associated with increased risk of a number of adverse maternal and neonatal health outcomes, yet research from developing countries is limited. We assessed the associations of maternal VDD during pregnancy with adverse health outcomes by synthesizing the literature from observational studies conducted in developing countries. Six electronic databases were searched for English-language studies published between 2000 and 2017. Thirteen studies from seven countries were included in the review. Prevalence of VDD ranged from 51.3% to 100%. Six studies assessed both maternal and neonatal outcomes, four studies assessed only maternal outcomes and three studies assessed only neonatal outcomes. Ten studies showed at least one significant association between VDD and adverse maternal and/or neonatal health outcomes including pre-eclampsia (*n* = 3), gestational diabetes mellitus (*n* = 1), postpartum depression (*n* = 1), emergency cesarean section delivery (*n* = 1), low birth weight babies (*n* = 4), small for gestational age (*n* = 2), stunting (*n* = 1). However most of these studies (*n* = 6) also showed no association with multiple health outcomes. Vitamin D assessment methods, criteria applied to define VDD, season and trimester in which studies were conducted varied considerably across studies. In conclusion, this study highlights the need to improve maternal vitamin D status in developing countries in an effort to support best maternal and child health outcomes across these regions. Future research should focus on more unified approaches to vitamin D assessment and preventative approaches that may be embedded into already existing antenatal care settings.

## 1. Introduction

Vitamin D deficiency (VDD), defined as 25 hydroxyvitamin D concentration (25OHD) below 50 nmol/L (20 ng/mL) [1] is recognized as a global health problem, affecting around one billion people across all ethnicities and age groups worldwide [2]. Low serum 25OHD has been described as completely preventable [3], and is considered an important public health priority, given the adverse health outcomes associated with its deficiency [4]. 

Vitamin D is produced endogenously following exposure of the skin to ultraviolet (UV) radiation, with only small proportions being derived from exogeneous dietary sources [5]. Yet, despite sufficient and stable sunny conditions across equatorial countries [2,6,7], studies have reported high prevalence of VDD in pregnant and lactating women [6,8] and infants [6,9,10] in such areas, ranging from 26–95% during pregnancy [11]. Women throughout Asia [12,13], the Middle East and Africa [13] have been consistently regarded as “high risk” for deficiency, with particularly high prevalence being observed throughout these continents. 

Vitamin D is a pleiotropic pro-hormone [14,15] that functions through an endocrine (regulation of calcium absorption) and autocrine (facilitation of gene expression) mechanism [14,15]. Once ingested or produced by the body via sunlight exposure, vitamin D is transported to the liver for hydroxylation to 25(OH)D, the main circulating form of vitamin D, and then to the kidney where the biologically active hormone form of vitamin D 1,25(OH)2D is produced. Maternal 25OHD can freely cross the human placenta whereby the placenta expresses vitamin D receptors (VDR) and the enzyme CYP27B1 that can convert 25(OH)D to its biologically active form 1,25-dihydroxyvitamin D (1,25OH2D). While it is beyond the scope of this review to provide detail regarding the plausible biological pathways through which vitamin D could influence neonatal health, during pregnancy, VDD has been associated with increased risk of a number of adverse maternal and child health outcomes. These include an increased risk of pre-eclampsia and gestational diabetes mellitus (GDM), increased production of maternal inflammatory cytokines [16], insulin resistance [17], primary caesarean section [17] and more recently, with high maternal body mass index (BMI) [18] and symptoms of postpartum depression [19]. For the offspring, increased risk of preterm birth [16], small for gestational age (SGA) babies [16], neonatal hypocalcemia [17], rickets in infancy [20], recurrent wheeze, asthma [17] and increased adiposity during childhood [18] have been documented outcomes associated with maternal VDD.

It is well established that women with dark skin pigmentation are at an increased risk of VDD [21], yet a range of lifestyle factors have also been identified as key contributors to obtaining sufficient vitamin D [22]. Increased time spent indoors and away from sunlight, liberal sunscreen use (and/or sun avoidance) secondary to skin cancer concern in some areas of the world, adoption of covered clothing due to religious, cultural or aesthetic reasons and global environmental pollution are all factors implicated in the increased prevalence of VDD [22]. Moreover, women living in low-income, developing countries are often unable to meet micronutrient demands of pregnancy due to a chronically poor diet [23]. Inadequate access to dietary sources of vitamin D such as oily fish and eggs or having a vegetarian diet, which is common in some regions throughout Asia, make attaining sufficient vitamin D via food sources increasingly difficult. Women with one or more of these lifestyle risk factors could be considered particularly vulnerable to VDD, which may impact upon their health and the health of their offspring. 

For women in developing countries specifically, although there has been recent research which has reported on the prevalence of VDD in such populations and in some cases on pregnancy complications [24,25,26,27], there has been no published systematic review assessing associations of maternal VDD with maternal and/or neonatal outcomes. In order to assist women to achieve optimal vitamin D levels during pregnancy via culturally suitable strategies, it is necessary to first investigate the impact of deficiency on health outcomes in “high risk” populations throughout the developing world. The aim of this review was to assess the association of maternal VDD with adverse maternal and neonatal outcomes by synthesizing the literature from studies conducted with women in developing countries. While not an overarching aim of the review, biological and lifestyle factors associated with increased risk of deficiency were also summarized when they were assessed.

## 2. Materials and Methods

### 2.1. Study Criteria

Observational studies which assessed associations between maternal VDD during pregnancy with maternal and/or neonatal complications or adverse health outcomes were reviewed. Studies which included either short term (during pregnancy, e.g., maternal pre-eclampsia) and/or long term (following pregnancy, e.g., maternal postpartum depression) health complications which were associated with VDD were eligible for inclusion. Further, studies which included well-established and medically diagnosed pregnancy and/or neonatal health complications (e.g., GDM, pre-eclampsia, caesarean section delivery, postpartum depression, low birth weight (LBW), SGA, rickets, osteoporosis etc.) as opposed to reported symptoms and/or sole risk factors for these conditions (or related morbidity), as study outcomes were eligible. 

Cross-sectional, longitudinal, cohort and ecological studies were eligible for inclusion while intervention studies were excluded as they would not allow for a true assessment of the impact of VDD on maternal and child health, in the absence of a specific treatment of the deficiency. We elected to exclude case-control studies as they are inherently susceptible to selection bias, incidence rates of maternal VDD could not be obtained via such studies and actual relative risk is unable to be determined [28]. They are also suitable particularly to assessment of rare diseases or outcomes [29] and as such were eliminated in the assessment of common pregnancy complications such as those included in the review. Non-human studies and those which did not include pregnancy and/or neonatal complications as either a primary or secondary outcome were excluded. There were no limitations placed on age due to known high rates of teenage/adolescent pregnancies across developing countries specifically [30], or timing of pregnancy and neonatal complications (e.g., trimesters, or diseases tracking into infancy or childhood). 

### 2.2. Search Strategy

A comprehensive search of the literature was conducted in July 2017 using the EBSCO host platform and included the MEDLINE, CINAHL, Health Source: Nursing/Academic Edition and Global Health databases. A separate search using the EMBASE platform was also conducted at the same time. Studies were limited by year (2000–2017) to English only and to human studies across all databases. The Preferred Reporting Items for Systematic Reviews and Meta-Analysis (PRISMA) guidelines were adhered to and formed the basis for methodology relevant to this review. A list of “developing countries” for searching purposes was derived from that previously defined by the Australian Minister of Foreign Affairs of the Department of Foreign Affairs and Trade (DFAT) for the Overseas Aid Gift Deduction, established in 1997 [31]. The search strategy combined the keywords Pregnan* OR Gestation* OR “Vitamin D” OR “serum 25-hydroxyvitamin D” OR “serum 25OHD” OR 25OHD OR “serum 25-hydroxyvitamin D (25OHD)” OR “hypovitaminosis D” OR cholecalciferol OR Deficien* OR Insufficien* OR “Developing Countr*” OR Brunei OR Cambodia OR “East Timor” OR “Timor Leste” OR Indonesia OR Lao OR Malaysia OR Myanmar OR Burma OR Phillippine OR Filipino OR Thailand OR Vietnam OR Turkmenistan OR Uzbekistan OR Maldives OR Mongolia OR Nepal OR Pakistan OR “Sri Lanka” OR Tajikistan OR Kazakhstan OR Korea OR Kyrgyzstan OR China OR India OR Afghanistan OR Bangladesh OR Bhutan OR Albania OR Armenia OR Azerbaijan OR Belarus OR “Bosnia & Herzegovina” OR Georgia OR Kosovo OR Macedonia OR Moldova OR Montenegro OR Serbia OR Turkey OR Ukraine OR Algeria OR Egypt OR Libya OR Morocco OR Tunisia OR Angola OR Benin OR Botswana OR “Burkina Faso” OR Burundi OR Cameroon OR “Cabo Verde” OR “Central African Republic” OR Chad OR Comoros OR Congo OR “Cote d’Ivoire” OR “Ivory Coast” OR Djibouti OR “Equatorial Guinea” OR Eritrea OR Ethiopia OR Gabon OR Gambia OR Ghana OR Guinea OR “Guinea-Bissau” OR Kenya OR Lesotho OR Liberia OR Madagascar OR Malawi OR Mali OR Mauritania OR Mauritius OR Mozambique OR Namibia OR Niger OR Nigeria OR Rwanda OR “St. Helena” OR “Sao Tome & Principe” OR Senegal OR Seychelles OR “Sierra Leone” OR Somalia OR “South Africa” OR “South Sudan” OR Sudan OR Swaziland OR Tanzania OR Togo OR Uganda OR Zambia OR Zimbabwe OR “Antigua & Barbuda” OR Belize OR “Costa Rica” OR Cuba OR Dominica OR “Dominican Republic” OR “El Salvador” OR Grenada OR Guatemala OR Haiti OR Honduras OR Jamaica OR Mexico OR Montserrat OR Nicaragua OR Panama OR “St. Lucia” OR “St. Vincent and the Grenadines” OR Argentina OR Bolivia OR Brazil OR Chile OR Colombia OR Ecuador OR Guyana OR Paraguay OR Peru OR Suriname OR Uruguay OR Venezuela OR Iran OR Iraq OR Jordan OR Lebanon OR “Syrian Arab Republic” OR “West Bank and Gaza Strip” OR Yemen OR “Cook Islands” OR Fiji OR Kiribati OR “Marshall Islands” OR Micronesia OR Nauru OR Niue OR Palau OR “Papua New Guinea” OR Samoa OR “Solomon Islands” OR Tokelau OR Tonga OR Tuvalu OR Vanuatu OR “Wallis & Futuna”.

### 2.3. Data Extraction

A data extraction template adapted from a previously published systematic review conducted by two of the authors [32] guided data extraction. Two researchers (PvP and JW) independently extracted the data addressing criteria consisting of study design and methodology, sample characteristics, approaches to measurement of vitamin D, criteria and prevalence of deficiency, health outcomes and biological or lifestyle characteristics associated with increased risk of deficiency. Any differences in the extraction and interpretation of data were resolved by discussion between the researchers.

### 2.4. Quality Assessment

Studies were assessed for quality using the McMaster University quality assessment tool [33]. As per the protocol, for each study, a rating scale assessed selection bias, study design, confounders, data collection and withdrawals and dropouts, with a rating of either weak, moderate or strong allocated to each component. Where detail of a component was missing a weak rating was given. An overall quality rating was given as either weak (two or more weak component ratings), moderate (less than four strong ratings and one weak rating) or strong (four strong ratings with no weak ratings). Two researchers (JW and EM) independently assessed study quality and any discrepancies in overall ratings were resolved by discussion.

## 3. Results

### 3.1. Study Selection

**Figure 1:**
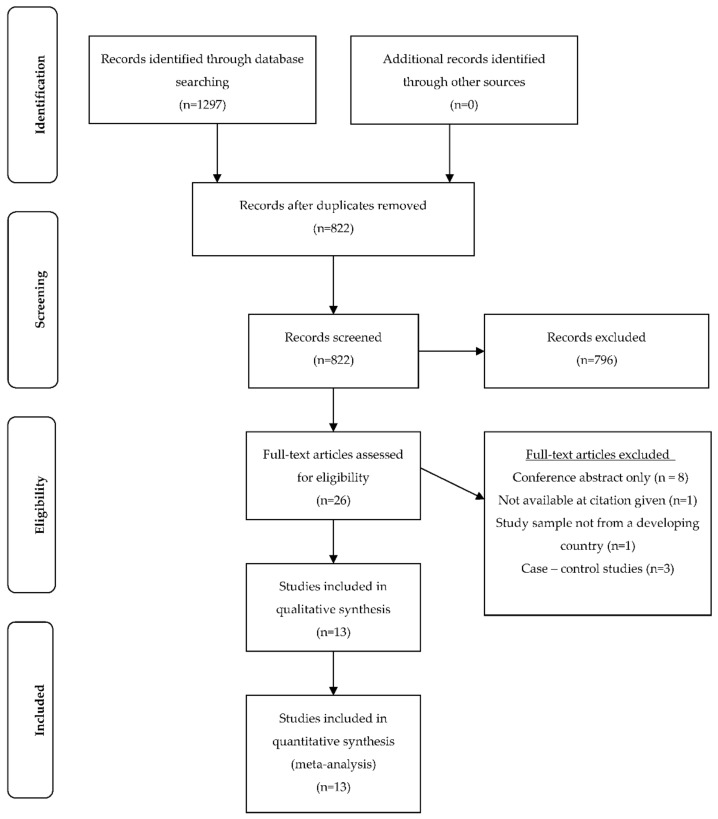
Flow diagram of included studies.

The EBSCO host platform search including MEDLINE, CINAHL, Health Source: Nursing/Academic Edition and Global Health retrieved 628 papers and the EMBASE platform search retrieved 669 papers. These were imported into an EndNote library and all duplicate studies were removed (*n* = 475), leaving a total of 822 papers. The title and abstract of each were reviewed by PvP and JW to assess eligibility and where insufficient detail was available in the title or abstract, full papers were assessed for inclusion. After reviewing the title and abstract of each paper, 26 studies were retained and the full-text papers were reviewed. Cross checking of titles and abstracts of excluded studies to ensure reliability of the screening process was conducted by JW. No additional papers were included via this process. Both PvP and JW independently assessed all full-text papers for inclusion eligibility. Level of agreement between the two researchers was high. Minor differences between researchers regarding the inclusion of papers were resolved by discussion. The bibliographies of papers for inclusion were hand searched but no further papers were included in the review via this process. A summary of the study selection process is presented in Figure 1. In total, 13 studies were included in the systematic review. A summary of each study included is presented in Table 1. For consistency all units of vitamin D measurement were converted to nmol/L (from ng/mL or otherwise) for study comparisons. Overall, ten out of the 13 studies included showed at least one significant association between VDD and adverse maternal and/or neonatal health outcomes. 

### 3.2. Setting and Participants

Overall the 13 studies represented data from seven different countries within Asia [24,26,34,35,36], the Middle East [25,37,38,39,40] and Africa [6,27]. Two studies were conducted in India [24,34], three in China [26,35,36], three in Turkey [37,38,41], two in Iran [25,40] and one in each of Pakistan [42], Kenya [6] and Nigeria [27]. Seven studies were conducted in the inpatient setting within large maternity hospitals [6,24,25,26,27,38,39] and four studies involved women who were recruited as outpatients, attending antenatal clinic bookings [34,37,40,41]. One study included women recruited through both of these settings [36] and the study conducted by Chen et al. assessed a sub sample of a large population-based birth cohort study in China [35]. Mean age of women across studies ranged from 22.5 years [6] to 31.3 years [27]. The number of women recruited ranged from 63 [6] to 3658 [35] and stage of gestation varied across studies ranging from early pregnancy at 13 weeks [37] to 39 weeks [38]. Eleven out of the 13 studies reported the trimesters in which women took part in the study and subsequently had their corresponding vitamin D levels assessed. These ranged from one study in the first trimester [37], two studies in the second trimester [40,41], two studies in both the second and third trimesters [6,36], four studies in just the third trimester [26,34,38,39] and two studies assessed women across all three trimesters of their pregnancy [27,35].

### 3.3. Vitamin D Assessment

Five different methods across studies were used to measure serum 25OHD levels. Six studies used enzyme-linked immunosorbent assay (ELISA) methods [6,24,25,26,38,41]. Each study showed one or more positive association between VDD and maternal and/or neonatal health outcomes. Three studies utilized radioimmunoassy (RIA) [34,35,40], with two studies showing positive associations between VDD and maternal GDM [40] and neonatal LBW and SGA [35]. Two studies used automated chemiluminescence immunoassay (CI) [36,39], both of which assessed maternal outcomes only and showed positive associations between VDD and maternal pre-eclampsia. One study used liquid chromatography tandem-mass spectrometry (LC/MS/MS) [37] and one used high-performance liquid chromatography (HPLC) [27] with both studies showing no significant associations between VDD and either maternal or neonatal outcomes. 

### 3.4. Vitamin D Deficiency, Criteria and Prevalence

With reference to the criteria used to define VDD and the reference values and cut-point scores for prevalence, approaches varied widely and were not dependent on the country in which the study was conducted. Likewise, prevalence of VDD differed widely within studies which employed the same techniques for assessment of vitamin D. There were varied classifications used to define VDD, inadequacy or insufficiency. Deficiency was defined as <50 nmol/L [24,27,34,35], <37.5 nmol/L [38] and as severe/moderate and/or mild in several studies [25,26,37,39,40,41], with variation as to how each was classified. 

Prevalence of combined deficiency and insufficiency (vs adequacy or sufficiency) ranged from 39.4% [27] to 76.5% [24] depending on the criteria used. Six studies further characterized the severity of deficiency into mild, moderate or severe [25,26,37,39,40,41] using a range of different cut-points.

When it was not combined with insufficiency, prevalence of VDD ranged from 51.3% [41] to 100% [26]. Overall the highest prevalence (>80%) were observed in Chinese women (100%) [26], Turkish women (95.6%) [37], women from Iran (89.4%) [40] and Pakistan (89.0%) [39]. Five studies showed prevalence of between 70 and 80% among their samples of Indian women [24], Chinese women [35,36], women from Iran [25] and women in Kenya [6], one study reported a prevalence of 67.0% in India [34] and the two studies conducted in Turkey showed prevalence of VDD between 50 and 60% [37,38]. The study conducted in Nigeria showed the lowest rates of VDD (39.4% when deficiency was combined with insufficiency and 29.0% deficiency alone) [27]. This study was also the only study to use HPLC for assessment of vitamin D and showed no significant association between VDD and maternal or neonatal outcomes. Likewise, there was wide seasonal variation in which studies were conducted. Six out of the 13 studies were conducted across all seasons or were “yearlong” and showed VDD prevalence ranging from 39.4% [27] to 95.6% [37]. Two studies with VDD prevalence of 55.4% [38] and 100% [26] did not report the season in which the study was conducted.

### 3.5. Maternal and Neonatal Outcomes

Ten out of the 13 studies (77%) included in this review showed significant associations between maternal VDD with either maternal and/or neonatal adverse health outcomes [6,24,25,26,35,36,38,40,41,42]. Overall six studies assessed the association of maternal VDD with both maternal and neonatal health outcomes [24,25,27,34,37,38], four studies assessed maternal outcomes only [36,40,41,42] and three studies [6,26,35] assessed only neonatal outcomes. A summary of assessed maternal and neonatal outcomes is shown in Table 2. 

Maternal VDD was shown to be associated with pre-eclampsia in three out of four studies [24,36,42] and GDM in one out of seven studies [40]. Associations with pregnancy and perinatal complications were found by Aydogmus et al. [38] (which was the only study to report grouped adverse maternal outcomes), postpartum depression in the only study by Gur et al. that assessed it [41] and abortion, emergency cesarean section delivery, oligohydramnios and polyhydramnios complication in one out of four studies [25]. Maternal VDD was not associated with premature or spontaneous rupturing of membranes (PROM and SROM) in two studies [27,38].

Neonatal outcomes adversely associated with maternal VDD were shown in five out of eight studies [6,24,25,35,38]. Maternal VDD was significantly associated with low birth weight (LBW) in four out of seven studies [24,26,35,38], small for gestational age (SGA) babies in two out of three studies [35,38] and stunting (but not wasting or BMI z-score) immediately post-delivery, which was only assessed as an outcome in the study by Toko et al. [6], and preterm birth in one out of five studies [6]. Among those outcomes which were not significantly associated with maternal VDD in any of the studies were Apgar score [24,25,27,37,38], NICU admission [24,38], neonatal death or stillbirth [27,37,38] or head circumference [25,26]. 

Each of the three studies that did not show any significant associations between VDD and adverse outcomes assessed both maternal and neonatal outcomes [27,34,37]. One study was conducted in each of Turkey [37], India [34] and Nigeria [27] where prevalence of VDD were 95.6%, 67.0% and 39.4% respectively. All three studies were conducted “yearlong” but recruited women at different stages of their pregnancies. Two out of the three studies [27,37] used the same criteria (serum 25OHD > 75 nmol/L) to define sufficient vitamin D, however criteria to define deficient vitamin D (e.g., severe, moderate, mild etc.) differed between the studies. 

### 3.6. Biological and Lifestyle Risk Factors

Seven out of the 13 studies assessed biological and lifestyle factors associated with increased risk of VDD, as part of their analyses [24,25,27,34,36,37,38]. In their study conducted in India, Ajmani et al. [24] found that factors which increased the risk of VDD were dark skin pigmentation, limited dairy and fish intake as sources of dietary vitamin D and limited outdoor activity. In a separate study in India, Farrant et al. [34] found that the risk of VDD increased in the autumn and winter months. Likewise, in Turkey it was reported that the risk of deficiency significantly increased in the winter months and in women who did not use multivitamins or with skin covering clothing [37]. In China, maternal age ≥35 years, pre-pregnancy BMI ≥25 kg/m^2^ and nulliparity were found to increase the risk of maternal VDD [36]. The remaining three studies showed no association between biological and lifestyle risk factors and maternal VDD [25,27,38].

### 3.7. Study Quality 

A summary of the quality of each study is presented in Table 3. All studies were given weak global ratings for study quality, defined as two or more weak ratings for individual study components including selection bias, study design, confounders, blinding, data collection methods and dropouts. Overall ratings for the 13 studies are presented in Table 4. Data collection methods were strong across all studies (13/13) while quality of study selection of participants and withdrawals and dropouts were varied considerably. Study design, blinding and adjustment for confounding variables were rated weak for the majority of studies (13/13; 11/13; 10/13). Overall, four out of the 13 studies adjusted for some confounding variables in their analyses [6,35,36,42]. All four studies adjusted for maternal age and either maternal pre-pregnancy BMI or maternal weight, three studies adjusted for gestational age [6,35,42], two studies adjusted for season [35,36] and one study adjusted for maternal physical activity [42]. 

## 4. Discussion

This systematic review is the first to summarize the available literature of observational studies on the impact of maternal VDD on adverse health outcomes for the mother and neonate, in developing countries. It is also the first systematic review to focus solely on this issue in low-income regions. We identified 13 studies that met the inclusion criteria, highlighting the paucity of research assessing this issue among “high risk” women throughout these regions. However, consistent with previous literature on maternal vitamin D status in other regions [43], reported prevalence rates of maternal VDD in this review from studies conducted across Asia, the Middle East and Africa were high, between 70–80% and as high as 100% in the one study conducted in China [26]. Notwithstanding the limited available data, the majority of these studies also showed that maternal VDD was associated with at least one maternal pregnancy complication and/or with complications related to neonatal growth.

Given the 13 included studies in this review were conducted across several countries and multiple continents, this suggests that VDD and its associated adverse health outcomes for women and children, are widespread. These findings are perhaps not surprising given that among developing countries, malnutrition and the burden of disease is high in relation to reproductive, maternal, newborn and child health [44]. Global data have consistently reported associations between maternal VDD with serious pregnancy complications including pre-eclampsia, GDM, infection and cesarean section delivery [45], which in part are consistent with findings from this review. Importantly, GDM increases the risk of pre-eclampsia developing later in pregnancy [46], which along with infection, are independently associated with increased risk of maternal mortality [47]. Moreover, hypertensive disorders in pregnancy are among the major causes of maternal death in developing countries, as reported by the World Health Organization (WHO) [48]. Despite rates of maternal mortality having declined over the last 10 years in some regions of Asia and Northern Africa [49], compared to in developed countries, maternal mortality rates for women in developing countries are 14-fold higher [50]. Factors which indirectly increase the risk of maternal mortality such as maternal VDD should be a consideration for public health initiatives, focused on improving adequacy and standards of antenatal healthcare in developing countries, to best support the health and survival rates of women during their pregnancies. 

It is well-established that neonatal vitamin D status can be determined directly by measuring circulating maternal 25(OH)D [3] and there is evidence to support and association between maternal 25OHD levels with adverse health outcomes. In this review, we identified five studies that reported significant associations between maternal VDD and LBW, SGA and stunting. Globally it is estimated that 23% of children under 5 years are stunted [51] and 15% of newborns have LBW [51] and combined, Africa and Asia account for almost 100% of the global burden of stunting [51]. Reductions in the prevalence of childhood stunting is a key target of the United Nations Sustainable Development Goals for 2030 [50] and addressing the issue of maternal and neonatal VDD should be a key component of action across developing countries in assisting achieving these global targets.

Children in developing countries born to breastfeeding mothers who are vitamin D deficient face a double burden in relation to risk of impaired growth and related morbidity. Despite exclusive breastfeeding in the first six months of life being recommended as the primary source of newborn feeding and nutrition [52], breast milk is a poor source of vitamin D [53,54]. Moreover, infants born with VDD, and/or who are exclusively breastfed with limited sun exposure, are at increased risk of rickets in the first year of life [55,56]. While supplementing breastfeeding with vitamin D can effectively assist in managing infant VDD, this practice occurs predominately in only high-income countries [57,58]. Public health strategies to ensure children attain sufficient vitamin D are therefore key to supporting a healthy start to life, healthy growth trajectories and a reducing the risk of stunting and infant rickets. 

Ten of the 13 studies included in this review found significant associations between maternal VDD and adverse health outcomes, yet many of these also found that VDD did not impact specific maternal and/or neonatal complications. In the wider literature including developed countries, methodological flaws have been identified as contributing to the inconsistent associations [59,60]. Similarly, in this review there was significant variation and heterogeneity in methodological approaches employed across studies with noticeable difference in the methods used to assess serum 25OHD concentrations, the stage during pregnancy at which it was measured, the criteria used to define deficiency or insufficiency and the trimester of pregnancy in which women took part in the studies. Seasonal differences and length of the study period also differed markedly. Such differences make drawing generalizable conclusions from both individual studies and the combined data difficult. Moreover, while ratings for study components varied in their quality within and across studies, overall study quality was poor. Just four of the studies adjusted for important confounders, known to impact maternal vitamin D and the various health outcomes assessed. Given there are well-established risk factors for VDD including increased BMI [1], adjustment for maternal pre-pregnancy BMI for example, and other confounders, should be ensured in future, along with similar studies assessing associations of VDD with pregnancy-related health outcomes. Further, investigation and assessment of the adoption of more unified approaches to vitamin D measurement, which are practically suitable across developing countries, should be a consideration for future research [61]. This would benefit planning, design and development of appropriate RCTs and culturally suitable preventative public health strategies targeting maternal VDD and associated health outcomes.

An important consideration in design of potential cohort studies and interventions in resource poor countries is the cost associated with assessing vitamin D status, and the approach used to measure serum 25OHD levels. Currently there are a number of different methods used to quantify serum 25OHD which has led to considerable variation in circulating levels across different assays and hampered attempts to develop evidence-based criteria for defining VDD, insufficiency and sufficiency. At present the LC-MS/MS-based assays are considered the reference method for measuring 25OHD [62] but are unlikely to be routinely available in many developing countries. To overcome issues related to different 25OHD assays, the vitamin D Standardization Program (VDSP) was developed in 2010 by the National Institutes of Health Office of Dietary Supplements, the Centers for Disease Control and Prevention (CDC), the National Institutes of Standards and Technology (NIST) and Ghent University, with the aim to standardize the laboratory measurement of serum 25OHD [63]. It is, therefore, recommended that all future studies ensure that serum 25OHD levels are assessed at certified laboratories to the standard reference method developed by NIST and Ghent University [63]. 

Currently the WHO does not recommend routine screening or supplementation of vitamin D during pregnancy [64], on the premise that there are a lack of high-quality evidence from RCTs providing sufficient rationale and safety for doing so. While there have been limited interventions to date which have targeted the improvement of pregnancy outcomes in relation to VDD in developing countries, of those available, most have largely focused on supplementation programs and reported mixed results [13,65]. Alongside supplementation, lifestyle factors and cultural differences should be considered when planning future interventions in low-income settings. While not an overarching aim of this systematic review, some studies did assess factors associated with maternal VDD. Among these, infrequent outdoor activity or wearing covered clothing for cultural or religious purposes (thereby reducing sunlight exposure), limited intake of dietary sources of vitamin D, and being overweight or obese pre-pregnancy were found to be associated with and increased risk of low maternal vitamin D status. Results from other studies have confirmed that these factors are consistent predictors of VDD [22,66]. Thus, it is likely that the combination of supplementation and food fortification where appropriate, with lifestyle modification (e.g., safe sun exposure) will provide the best approaches to attaining sufficient vitamin D [22]. Relief of poverty and an improved food supply will assist the long-term solution to this key public health issue. 

In order to design and implement successful intervention strategies focused on improving maternal vitamin D status, the structure of antenatal healthcare systems across developing countries and the resources and services available to women during pregnancy must be thoroughly understood and carefully considered. While all studies in this review were conducted within large maternity hospitals, obstetric departments or outpatient clinics, many women in resource-poor countries do not have access to such antenatal services, making opportunity for assessment of vitamin D and intervention during pregnancy particularly challenging. Understanding available models of care throughout remote regions is key to maximizing health promotion opportunities. From a public health perspective, women in these areas should be considered equally to those with better access to more sophisticated obstetric and antenatal services, as they are likely to comprise a large proportion of the vitamin D deficient population. Moreover, the preconception period may be a more viable window of opportunity in which to approach this public health issue, by reducing the number of women entering pregnancy with VDD. Future research might explore the preconception period as a preferred time in which to intervene and focus on preventative lifestyle approaches aimed at adolescents and young women, prior to pregnancy. 

A limitation of this review was the exclusion of a meta-analysis to combine the results of individual studies. A meta-analysis would enable improving the estimates of the effect size of observed associations, thereby strengthening the conclusions and inferences drawn from the data. However, the wide methodological variation and heterogeneity in methods used to assess vitamin D and classify deficiency across studies did not allow for pooling of results. Notwithstanding, this review was the first to synthesize the literature from developing countries specifically, assessing associations of VDD with pregnancy and neonatal complications. This has been a key step in further understanding where future work might be directed, in an effort to improve maternal and child health outcomes in regions where adequate nutrition support services for women during pregnancy are currently limited or lacking. 

## 5. Conclusions

Pregnant women and neonates throughout the developing world represent a population group highly susceptible to VDD and associated adverse health outcomes. This review showed that VDD during pregnancy is a widespread issue that can adversely affect the health of both women and children in developing countries. However, current methods to assess VDD differ widely and a more unified approach would assist informing the development of suitable approaches to tackling this issue. Addressing the impact of maternal and neonatal VDD through high-quality, rigorous and culturally suitable research, is key to informing well-designed RCTs targeting this issue. Future research should focus on strategies which include biological and lifestyle factors and preventative approaches that may be embedded into already existing antenatal care settings.

## Figures and Tables

**Table 1:** Description of included studies.

Study	Design	Recruitment	Sample	Serum Vitamin D Measurement	Criteria for Vitamin D Deficiency and Reported Prevalence	Outcomes	Risk Factors
Ajmani et al., 2016 India [24]	Prospective cohort	Approached/screened: not reported Enrolled: not reported Complete data: *n* = 200 burka-clad women Setting: antenatal clinic and inpatients in antenatal ward at Kasturba Hospital, Delhi Season: all (year-long)	Age: mean 24.8 years Demographics: 64.5% multigravida, 44% dark complexion, 36% low SES, 64% illiterate, 2.5% graduate level educated Trimester: not reported Exclusion criteria: non-burka clad, age <18 years or >40 years, history of liver/renal disease, osteoporosis or rheumatoid arthritis, antitubercular or antiepileptic treatment in last 6 months, taking vitamin D supplements	ELISA	Criteria *: Deficiency < 50 nmol/L Inadequacy 50–75 nmol/L Adequate > 75 nmol/L Prevalence of deficiency: Deficient *n* = 75 (37.5%) Inadequate *n* = 78 (39%) Adequate *n* = 47 (23.5%)	Maternal: 7.5% women diagnosed with pre-eclampsia, significant correlation between VDD and pre-eclampsia (*p* = 0.001) Neonatal: 9.5% LBW babies, significant correlation between VDD and LBW (*p* = 0.0001) No correlation: VDD and GDM, LSCS, bony abnormality, Apgar score, premature birth or NICU admission	Dark skin complexion, limited outdoor activity, low dairy intake, low fish intake (*p* < 0.05)
Ates et al., 2016 Turkey [37]	Prospective cohort	Approached/screened: *n* = 286 Enrolled: *n* = 266 (93%) Complete data: *n* = 229 (86%) Setting: first antenatal appointment at outpatient clinic of Obstetrics and Gynecology, Bezmialem Vakif University, Istanbul Season: 48.9% summer (May–October), 51.1% winter (November–April)	Age: mean 29.5 years Demographics: 64.5% primigravida, mean BMI 25.3 kg/m^2^, 61.3% covered dress, 63.1% multivitamin use, 6.6% smoking, 46.9% ≥ 9 years education Trimester: first Exclusion criteria: thyroid, parathyroid or adrenal disease, hepatic or renal failure, metabolic bone disease, medication affecting vitamin D metabolism, multiple pregnancy, taking vitamin D supplements	LC-MS/MS	Criteria *: Severe deficiency < 25 nmol/L Mod deficiency 25–47.5 nmol/L Mild deficiency 50–72.5 nmol/L Adequate > 75 nmol/L Prevalence of deficiency: Severe *n* = 105 (45.9%) Mod *n* = 83 (36.2%) Mild *n* = 31 (13.5%) Adequate *n* = 10 (4.4%)	Maternal: 53.8% women with severe VDD had vaginal delivery, compared with 32.7% as primary caesarean section (*p* = 0.018) No correlation: VDD and GDM, pre-eclampsia, gestational hypertension, preterm birth, SGA, intrauterine fetal death, congenital malformation, birth weight or Apgar score	Covered dress, non-multivitamin use, winter (*p* < 0.05)
Aydogmus et al., 2014 Turkey [38]	Prospective cohort	Approached/screened: *n* = 180 Enrolled: *n* = 152 (84%) Complete data: *n* = 148 (97%) Setting: inpatients at Izmir Katip Celebi University Ataturk Training and Research Hospital, Izmir Season: not reported	Age: mean 24.4 years Demographics: not reported Trimester: third Exclusion criteria: taking vitamin D supplements, multiparity, disease affecting vitamin D and calcium metabolism, medications for chronic disease	ELISA	Criteria *: Deficient < 37.5 nmol/L Insufficient 37.5–72.5 nmol/L Sufficient > 75 nmol/L (grouped for analysis Deficient < 37.5 nmol/L; Other ≥ 37.5 nmol/L) Prevalence of deficiency: Deficient: *n* = 66 (44.6%) Other *n* = 82 (55.4%)	Maternal: 39.9% women with VDD had poor pregnancy outcomes compared with 23.2% of women without VDD (*p* = 0.001), VDD increased risk of perinatal complications (OR 4.30; 95% CI 1.85–9.99) Neonatal: 16.7% SGA neonates born to mothers with VDD compared with 4.9% neonates born to mothers without VDD (*p* = 0.007), VDD increased risk of SGA (OR 4.5; 95% CI 1.41–15.78); mean birthweight significantly lower for neonates born to mothers with VDD (3187.6 ± 495.5 g) compared with those born to mothers without VDD (3268.1 ± 477.1 g) (*p* = 0.02) No correlation: VDD and mode of delivery, post maturity, GDM, maternal anemia, hypertension, pre-eclampsia, cholestasis, oligohydraminos, fetal distress, still birth, preterm labor, PPROM, Apgar scores, prolonged hospitalization, mortality, NICU admission or macrosomia	No significant associations
Chen et al., 2015 China [35]	Prospective cohort	Approached/screened: *n* = 4358 (sub-sample of a population-based cohort study *n* = 16,766) Enrolled: *n* = 3658 (84%) Complete data: 3658 (100%) Setting: women recruited to the larger China-Anhui Birth Cohort study from six major cities of Anhui province Season: all (year-long), 36.7% spring, 22.5% summer, 20.6% autumn, 20.2% winter	Age: mean 27.5 years Demographics: 96.0% nulliparous, 45.2% low income, 75.3% healthy BMI, 16.5% multivitamin use Trimester: all, 35.1% first, 62.0% second, 2.9% third Exclusion criteria: multiple pregnancy, abortion	RIA	Criteria *: Deficient < 50 nmol/L Insufficient 50–74.75 nmol/L Sufficient ≥ 75 nmol/L Prevalence of deficiency: Deficiency *n* = 1405 (38.4%) Insufficiency *n* = 1289 (35.2%) Sufficient *n* = 964 (26.4%)	Maternal: not assessed Neonatal: 16.01% SGA neonates born to mothers with VDD compared with 5.59% born to mothers with vitamin D insufficiency and 2.80% with sufficient vitamin D (*p* < 0.001); compared to sufficiency, maternal VDD increased risk of SGA (RR 6.47; 95% CI 4.30–9.75) and insufficiency (RR 2.01; 95% CI 1.28–3.16) (*p* < 0.001); 4.98% LBW neonates born to mothers with VDD compared with 1.32% born to mothers with vitamin D insufficiency and 0.41% with sufficient vitamin D (*p* < 0.001); VDD increased risk of LBW (RR 12.31; 95% CI 4.47–33.89) (*p* < 0.001). Adjusted for: Pre-pregnancy maternal BMI, maternal age, season and gestational week	Not assessed
Farrant et al., 2009 India [34]	Prospective cohort	Approached/screened: *n* = 1539 Enrolled: *n* = 830 (54%) Complete data: *n* = 674 (81%) Setting: women attending antenatal clinic at Holdsworth Memorial Hospital, Mysore Season: all (year-long)	Age: mean 23.7 years Demographics: mean BMI 23.4 kg/m^2^, women supplemented at recruitment (*n* = 156) with vitamin D as part of routine management, no information available at 30 weeks Trimester: third Exclusion criteria: not reported	RIA	Criteria: Hypovitaminosis < 50 nmol/L Adequate > 50 nmol/L Prevalence of deficiency: Hypovitaminosis *n* = 372 (67%) Adequate: *n* = 187 (33%)	No correlation: VDD and GDM, birthweight, impaired fetal growth	Autumn/winter (*p* < 0.05)
Gbadegesin et al., 2016 Nigeria [27]	Prospective cohort	Approached/screened: not reported Enrolled: *n* = 461 Complete data: *n* = 461 (100%) Setting: maternity unit of the Lagos State University Teaching Hospital, Ikeja and women of mixed ethnicity, social class and religion Season: all (year-long)	Age: mean 31.3 years Demographics: mean parity 1.16 Trimester: all Exclusion criteria: multiple pregnancy, previous medical condition (hypertension, renal disease, diabetes), taking vitamin D supplements, elevated BP	HPLC	Criteria *: Deficiency < 50 nmol/L Insufficiency 52.5–75 nmol/L Adequate > 75 nmol/L Prevalence of deficiency: Deficiency *n* = 134 (29.0%) Insufficiency *n* = 48 (10.4%) Adequate *n* = 279 (60.6%)	No correlation: VDD and preeclampsia, SROM, anemia, GDM, preterm delivery, mode of delivery, Apgar score or stillbirth	No significant associations
Gur et al., 2014 Turkey [41]	Prospective cohort	Approached/screened: *n* = 687 Enrolled: *n* = 208 (30%) Complete data: *n* = 189 at 1/52 (91%); *n* = 184 at 6/52 (88%); *n* = 179 at 6/12 (86%) Setting: women attending routine antenatal reviews at Sifa University Bornova Health Research and Application Hospital, Izmir Season: summer/autumn	Age: mean 28.5 years Demographics: mean BMI 26.5 kg/m^2^, 7.6% women supplemented with vitamin D ≥ 3 days per week and 84.6% supplemented daily, all women Caucasian and native Turkish speaking Trimester: second Exclusion criteria: unmarried, unplanned pregnancy, BMI < 20 or >30 kg/m^2^, smoker, diagnosed psychiatric illness, pre-diagnosed medical condition, parity > 3, education level < 8 years, multiple birth, employed, annual income < US $450, fetal death, complex delivery, newborn with anomaly, postpartum bleeding or hysterectomy	ELISA	Criteria *: Severe deficiency < 25 nmol/L Mild deficiency 25 nmol/L–50 nmol/L Normal ≥ 50 nmol/L Prevalence of deficiency: Severe: *n* = 23 (11%) Mild *n* = 84 (40.3%) Normal *n* = 101 (48.5%)	Maternal: 21.1%, 23.2% and 23.7% women had PPD at week 1, 6 and 6 months respectively; significant negative correlation (r = −0.2, −0.2, −0.3) between vitamin D levels and Edinburgh Postnatal Depression Scale (EPDS) score at each of the three time points; mean vitamin D level was significantly different between women with and without PPD at each of the three time points (*p* = 0.003, *p* = 0.004 and *p* < 0.001 respectively) Neonatal: not assessed	Not assessed
Hossain et al., 2010 Pakistan [39]	Cross-sectional	Approached: not reported (all women admitted to the labor suite for delivery during the study period were deemed eligible) RR: not reported Complete data: *n* = 75 Setting: delivery at Dow University of Health Sciences and Civil Hospital, Karachi Season: spring	Age: mean 26.0 years Demographics: mean BMI 27 kg/m^2^, mean parity 2.2, 26% covering arms, hands heads, 76% covering face Trimester: third Exclusion criteria: not reported	CI	Criteria *: Severe deficiency < 25 nmol/L Mod deficiency 27.5–50 nmol/L Mild deficiency 52.5–60 nmol/L Adequate > 60 nmol/L Prevalence of deficiency: Severe: *n* = 34 (45%) Mod: *n* = 20 (27%) Mild: *n* = 13 (17%) Adequate: *n* = 8 (11%)	Maternal: compared with women in the highest tertile for vitamin D, women in the lowest tertile and mid-tertile were more likely to meet criteria for pre-eclampsia and gestational pre-hypertension (OR 2.28; 95% CI 0.35–23.28) and (OR 19.27; 95% CI 1.96–188.92 respectively); vitamin D levels were inversely correlated with maternal mean arterial pressure (r = 0.029) (*p* = 0.020) Neonatal: not assessed in relation to maternal vitamin D Adjusted for maternal age, level of exercise, maternal weight, birthweight and gestational age	Not assessed
Maghbooli et al., 2008 Iran [40]	Cross-sectional	Approached/screened: not reported Enrolled: *n* = 741 Complete data: *n* = 579 Setting: referral to five university hospital clinics of the Tehran University of Medical Sciences during the first half of pregnancy Season: not reported	Age: mean 27.4 years Demographics: mean BMI 26.4 kg/m^2^ Trimester: second Exclusion criteria: prenatal diabetes	RIA	Criteria: Severe deficiency < 12.5 nmol/L Mild deficiency 12.5–24.9 nmol/L Mod deficiency 25–34.9 nmol/L Sufficiency > 34.9 nmol/L Prevalence of deficiency: Severe *n* = 201 (27.1%) Mild *n* = 118 (15.9%) Mod *n* = 344 (46.4%) Sufficient *n* = 78 (10.5%)	Maternal: 52% women diagnosed with GDM, mean vitamin D significantly lower in women with GDM (16.49 ± 10.44 nmol/L) compared with non-GDM women (22.97 ± 18.25 nmol/L) (*p* = 0.009), prevalence of severe VDD was significantly higher in women with GDM (44.2%) compared with non-GDM women (23.5%) (*p* = 0.011) Neonatal: not assessed	Not assessed
Pirdehghan et al., 2016 Iran [25]	Cross-sectional	Approached/screened: not reported Enrolled: not reported Complete data: *n* = 200 Setting: admission to hospital delivery room for natural delivery, caesarean section or abortion at Shahid Sadoughi hospital Season: autumn/spring	Age: mean 26.7 years Demographics: all women nulliparous, 48.7% diploma/university educated, 97.5% housewives, 38.5% women taking multivitamins containing vitamin D during pregnancy, Trimester: not reported Exclusion criteria: pre-existing medical conditions (renal or bone disorders), medication influencing calcium or vitamin D metabolism. multiparity	ELISA	Criteria *: Severe deficiency < 25 nmol/L Moderate deficiency 25–50 nmol/L Mild deficiency 52.5–75 nmol/L Adequate 75–125 nmol/L Upper normal/toxic > 125 nmol/L Prevalence of deficiency: (figures reported in text) Severe 12.5% Deficiency 60%	Maternal: mean vitamin D significantly higher in natural or elective caesarean section women compared with abortion and emergency caesarean section women (*p* = 0.040); VDD associated with risk of abortion 3.1 (1.39–6.8) which was higher in severe deficiency women compared with VDD women (*p* = 0.045), mean vitamin D significantly lower in women with oligohydramnios or polyhydramnios complication (13.9 + 9.5 and 20.6 + 10.8 respectively) (*p* = 0.045) No correlation: VDD and preeclampsia, PROM, GDM, birth weight, birth length, head circumference or Apgar score	No significant associations
Song et al., 2012 China [26]	Cross-sectional	Approached/screened: not reported Enrolled: not reported Complete data: 70 Setting: delivery at 306 Hospital of PLA in Beijing from surrounding communities of the Beijing urban area Season: spring	Age: 29.9 (±0.3) years Demographics: Mean weight: 73.9 kg, pregravid range 0–3 Trimester: third Exclusion criteria: multiparity, taking calcium and/or vitamin D supplements, pre-existing medical conditions (hypertension, renal disease, pre-gestational diabetes)	ELISA	Criteria: Severe deficiency < 25 nmol/L Mild deficiency 25– < 50 nmol/L Insufficiency 50– < 75 nmol/L (21–29 ng/mL) Sufficiency ≥ 75 nmol/L (grouped for analysis Deficient < 25 nmol/L and other ≥ 25 nmol/L Prevalence of deficiency: Severe *n* = 38 (54.5%) Mild *n* = 25 (35.7%) Insufficient *n* = 7 (10.0%) Sufficient *n* = 0 (0%)	Maternal: not assessed Neonatal: significant correlation between maternal vitamin D and newborn length (r = 0.247) (*p* = 0.039); compared with women who had vitamin D ≥ 25 nmol/L, birth weight (3633.1 g) and length (51.0 cm) of newborns were significantly lower in women with vitamin D < 25 nmol/L (3386 g and 50.2 cm respectively) (*p* = 0.015, *p* = 0.037) No correlation: VDD or head circumference	Not assessed
Toko et al., 2016 Kenya [6]	Longitudinal	Approached/screened: *n* = 99 RR: not reported Complete data: *n* = 63 (baseline data used) (64%) Setting: women residing within a 10 km radius of Chulaimbo Sub-district hospital in Kisumu County Season: dry season	Age: mean 22.5 years Demographics: mean BMI 22.9 kg/m^2^ Trimester: second and third Exclusion criteria: more than 26 weeks gestation, HIV infected, residing >10 km from the hospital	ELISA	Criteria: Deficiency < 50 nmol/L Insufficiency 50–75 nmol/L Sufficiency > 75 nmol/L (grouped for analysis low < 50 nmol/L and adequate ≥ 50 nmol/L) Prevalence of deficiency: Deficient *n* = 13 (20.6%) Insufficient *n* = 32 (50.8%) Sufficient *n* = 19 (28.6%)	Maternal: not assessed Neonatal: newborns more likely to have stunted growth at birth when born to mothers with deficient vitamin D (RR 4.4 (CI 1.0–18.6) (*p* = 0.04) and more likely to be born preterm (<37 weeks) (RR 5.4 (CI 1.1, 25.3) (*p* = 0.03) Adjusted for: maternal age, gestational age at delivery and maternal BMI No correlation: VDD and wasting or BMI z-score	Not assessed
Xin et al., 2017 China [36]	Prospective cohort	Approached/screened: not reported Enrolled: *n* = 13,806 Complete data: *n* = 11,151 (81%) Setting: routine visit to antenatal care clinic and delivery at the Wuxi Maternity and Child Health Hospital Season: 28.4% winter, 18.5% spring, 22.7% autumn, 30.4% summer	Age: mean 27.3 years Demographics: 88.9% nulliparous, 9.2% BMI ≥ 25 kg/m^2^, 96% GA at delivery ≥ 37 weeks Trimester: second and third Exclusion criteria: taking calcium and/or vitamin D supplements, pre-existing medical conditions (hypertension, renal disease, pre-gestational diabetes), fetal anomalies	CI	Criteria: Deficiency *<* 50 nmol/L Sufficiency > 50 nmol/L Prevalence of deficiency: Deficient *n* = 8799 (78.9%) Sufficient *n* = 2352 (20.8%)	Maternal: 1.2% pre-eclampsia, significant difference in incidence of severe pre-eclampsia in pregnant women with VDD (<50 nmol/L) (*n* = 123; 1.4%) compared with sufficiency (≥50 nmol/L) (*n* = 16; 0.6%) (*p* = 0.002), women with VDD were more at risk of developing severe pre-eclampsia compared with women who were vitamin D sufficient (OR: 3.16; 95% CI: 1.77–5.65) (*p* = 0.000) Adjusted for: pre-pregnancy BMI, maternal age, parity and season of blood sampling Neonatal: not assessed	Age ≥ 35 years, pre-pregnancy BMI ≥ 25 kg/m^2^, nulliparity (*p* < 0.05)

* Vitamin D unit of measurement converted from ng/mL to nmol/L. Abbreviations: VDD—vitamin D deficiency; BMI—Body Mass Index; CI—automated chemiluminescence immunoassay; ELISA—enzyme-linked immunosorbent assay; GDM—gestational diabetes mellitus; LC-MS/MS—liquid chromatography tandem-mass spectrometer; LSCS—lower segment caesarean section; NICU—neonatal intensive care unit; OR—odds ratio; PPD—postpartum depression; RIA—radioimmunoassay; SGA—small for gestational age; PROM—premature rupture of membrane; SROM—spontaneous rupture of membranes.

**Table 2:** Associations of VDD with reported maternal and neonatal outcomes across studies.

Maternal Outcomes													
Study	PE	GDM	Anemia	GHTN	OHD	Mode of Delivery *	Cholestasis	PPO	PH	MMAP	PPD	PC	PROM/SROM
Ajmani et al. (2016) India [24]	+	-				-							
Ates et al. (2016) Turkey [37]	-	-		-		+							
Aydogmus et al. (2014) Turkey [38]	-	-	-	-	-	-	-	+	-			+	-
Chen et al. (2015) China [35]													
Farrant et al. (2009) India [34]		-											
Gbadegesin et al. (2016) Nigeria [27]	-	-	-			-							-
Gur et al. (2014) Turkey [41]											+		
Hossain et al. (2010) Pakistan [39]	+			+						+			
Maghbooli et al. (2008) Iran [40]		+											
Pirdehghan et al. (2016) Iran [25]	-	-			+								-
Song et al. (2012) China [26]													
Toko et al. (2016) Kenya [6]													
Xin et al. (2017) China [36]	+												
**Neonatal Outcomes**													
**Study**	**Macro.**	**Stunted Growth** *	**Preterm Birth**	**W z-Score**	**Still Birth/IFD**	**NICU Admit**	**Apgar Score**	**Post MB**	**HC**	**CM** *	**Birth Weight** *	**Birth Length**	**SGA**
Ajmani et al. (2016) India [24]			-			-	-			-	+		
Ates et al. (2016) Turkey [37]			-		-		-			-	-		-
Aydogmus et al. (2014) Turkey [38]	-		-		-	-	-	-			+		+
Chen et al. (2015) China [35]											+		+
Farrant et al. (2009) India [34]		-									-		
Gbadegesin et al. (2016) Nigeria [27]			-		-		-						
Gur et al. (2014) Turkey [41]													
Hossain et al. (2010) Pakistan [39]													
Maghbooli et al. (2008) Iran [40]													
Pirdehghan et al. (2016) Iran [25]							-		-		-	-	
Song et al. (2012) China [26]									-		+	+	
Toko et al. (2016) Kenya [6]		+	+	-									
Xin et al. (2017) China [36]													

Note that +: significant association between VDD and the outcome in question; -: no significant association. When neither + or - is given indicated that outcome was not assessed. VDD—vitamin D deficiency; PE—pre-eclampsia; GDM—gestational diabetes mellitus; GHTN—gestational hypertension; OHD—oligohydramnios; PROM—premature rupture of membrane; SROM—spontaneous rupture of membranes; IFD—Intrauterine Fetal Death; NICU—neonatal intensive care unit; HC—head circumference; CM—congenital malformation SGA—small for gestational age; PH—prolonged hospitalization; Macro—macrosomia; Post MB—post mature birth; MMAP—mean maternal arterial pressure; W z-score—wasting z score; PPO—poor pregnancy outcomes (grouped by study researchers); PPD—postpartum depression; PC—perinatal complications risk. * Grouped outcomes: Mode of Delivery (includes vaginal delivery and lower segment caesarean section), Birth weight (includes low birth weight), Congenital Malformation (includes Bony Abnormality), Stunted Growth (includes Impaired Fetal Growth), Still birth/IFD (includes mortality).

**Table 3:** Summary of study quality assessment ratings for included studies.

Study	Selection Bias	Study Design	Cofounders	Blinding	Data Collection Method	Withdrawals and Dropouts	Global Rating
Ajmani et al. (2016) India [24]	3	3	3	3	1	3	Weak
Ates et al. (2016) Turkey [37]	1	3	3	3	1	1	Weak
Aydogmus et al. (2015) Turkey [38]	2	3	3	3	1	1	Weak
Chen et al. (2015) China [35]	1	3	1	3	1	1	Weak
Farrant et al. (2009) India [34]	2	3	3	3	1	2	Weak
Gbadegesin et al. (2016) Nigeria [27]	3	3	3	3	1	3	Weak
Gur et al. (2014) Turkey [41]	3	3	3	2	1	1	Weak
Hossain et al. (2010) Pakistan [39]	3	3	3	3	1	3	Weak
Maghbooli et al. (2008) Iran [40]	2	3	3	3	1	3	Weak
Pirdehghan et al. (2016) Iran [25]	3	3	3	2	1	3	Weak
Song et al. (2012) China [26]	3	3	3	3	1	3	Weak
Toko et al. (2016) Kenya [6]	3	3	1	3	1	3	Weak
Xin et al. (2017) China [36]	2	3	1	3	1	1	Weak

1: Strong; 2: Moderate; 3: Weak.

**Table 4:** Summary of global ratings for study quality.

Quality Rating			
Quality Component	Strong (*n*)	Moderate (*n*)	Weak (*n*)
Selection bias	2/13	4/13	7/13
Study design	0/13	0/13	13/13
Confounders	3/13	0/13	10/13
Blinding	0/13	2/13	11/13
Data collection methods	13/13	0/13	0/13
Withdrawal and dropout	5/13	1/13	7/13
Global rating	0/13	0/13	13/13

1: Strong; 2: Moderate; 3: Weak. Global weak quality: two or more weak component ratings; moderate quality: less than four strong and one weak component ratings, high quality: four strong and no weak component ratings.
